# Numerical Analysis of Load Reduction in the Gliding Process Achieved by the Bionic Swan’s Webbed-Foot Structures

**DOI:** 10.3390/biomimetics10060405

**Published:** 2025-06-16

**Authors:** Fukui Gao, Xiyan Liu, Xinlin Li, Zhaolin Fan, Houcun Zhou, Wenhua Wu

**Affiliations:** 1 Aerospace Tecnology Institude, China Aerodynamics Research and Development Center, Mianyang 621000, China; gaofukui@alumni.nudt.edu.cn (F.G.); liuxiyan1992@mail.nwpu.edu.cn (X.L.); 2 Key Laboratory of Cross-Domain Flight Interdisciplinary Technology, China Aerodynamics Research and Development Center, Mianyang 621000, China; 3 Institute of Composite Materials and Strutures, Harbin Institute of Technology, Harbin 150080, China; lixinlin@hit.edu.cn; 4 China Aerodynamics Research and Development Center, Mianyang 621000, China

**Keywords:** cross-medium vehicles, swan’s water entry, global motion mesh, computational fluid dynamics, bionic design

## Abstract

Webbed-foot gliding water entry is a characteristic water-landing strategy employed by swans and other large waterfowls, demonstrating exceptional low-impact loading and remarkable motion stability. These distinctive biomechanical features offer significant potential for informing the design of cross-medium vehicles’ (CMVs’) water-entry systems. To analyze the hydrodynamic mechanisms and flow characteristics during swan webbed-foot gliding entry, the three-dimensional bionic webbed-foot water-entry process was investigated through a computational fluid dynamics (CFD) method coupled with global motion mesh (GMM) technology, with a particular emphasis on elucidating the regulatory effects of entry parameters on dynamic performance. The results demonstrated that the gliding water-entry process can be divided into two distinct phases: stable skipping and surface gliding. During the stable skipping phase, the motion trajectory exhibits quasi-sinusoidal periodic fluctuations, accompanied by multiple water-impact events and significant load variations. In the surface-gliding phase, the kinetic energy of the bionic webbed foot progressively decreases while maintaining relatively stable load characteristics. Increasing the water-entry velocity will enhance impact loads while simultaneously increasing the skipping frequency and distance. Increasing the water-entry angle will primarily intensify the impact load magnitude while slightly reducing the skipping frequency and distance. An optimal pitch angle of 20° provides maximum glide-skip stability for the bio-inspired webbed foot, with angles exceeding 25° or below 15° leading to motion instability. This study on webbed-foot gliding entry behavior provided insights for developing novel bio-inspired entry strategies for cross-medium vehicles, while simultaneously advancing the optimization of impact-mitigation designs in gliding water-entry systems.

## 1. Introduction

With the rapid advancement of marine science and the increasing diversity of human activities in the ocean, the operational efficiency of traditional single-medium vehicles has become insufficient to meet the demands of cross-domain air–sea missions [1]. In this context, the CMV has gradually emerged as a research hotspot [2]. The CMV, capable of freely transitioning between air and water, is regarded as a breakthrough technology for expanding the domains of aerial and marine exploration [3]. The CMV not only inherits the high mobility and rapid deployment capabilities of traditional unmanned aerial vehicles (UAVs) but also possesses the high stealthiness of underwater vehicles, demonstrating unique advantages that traditional UAVs cannot match [4].

The mission scenarios of CMVs encompass aerial and underwater navigation as well as water-entry and -exit movements [5]. The water-entry phase, in particular, involves the coupling of gas, liquid, and solid phases, characterized by high coupling, nonlinearity, and unsteadiness, making its mechanical properties extremely complex and posing significant challenges to the design and application of such vehicles [6,7]. Firstly, structural safety is a critical concern. Due to the 800-fold difference in density between water and air, the vehicle experiences intense impact loads during the instant of water entry, posing a major threat to the integrity of its structure and internal components [8,9]. Secondly, motion stability is equally crucial. The impact loads and pitching moments during water entry can affect the vehicle’s speed and attitude, potentially leading to imbalance, flipping, or capsizing. Therefore, designing a reliable and stable in-water motion strategy that can ensure a smooth transition of the vehicle to underwater operations has become an important design requirement for CMVs [10,11].

To address these challenges, researchers have drawn inspiration from nature, particularly waterfowls with air–water transition capabilities. These waterfowls, through precise perception and flexible control, can seamlessly switch between air and water, efficiently performing actions such as water entry and foraging while avoiding damage from water impact [12,13,14]. Currently, several bionic CMVs have been developed, but their adaptability, reliability, and robustness still fall far short of those of air–water amphibious birds [15,16]. Large waterfowl such as swans, with their significant weight, comparable to CMVs, and high maneuverability during water entry and exit, serve as ideal subjects for studying bionic cross-medium motion mechanisms [17]. During the water-entry process, the swan decelerates by gliding on the surface of the water using its webbed feet, avoiding the impact effect of water on its body. This method of water entry demonstrates the unique characteristics of a low impact load and high motion stability, which are the research targets of this paper.

Currently, the investigations of biological motion characteristics and fluid dynamics primarily employ three methods: experimentation [18,19], numerical simulation [20,21], and theoretical analysis [22,23]. Among these, numerical computation has become a crucial tool in bionics research due to its unique advantages in revealing flow-field evolution and the interaction mechanisms between organisms and fluids. In recent years, numerical simulations have achieved significant progress in the field of biological cross-medium motion: Huang et al. [24] systematically analyzed the motion patterns and mechanisms of cormorant webbed feet during water surface takeoff using the CFD software FLUENT 2020 R2, proposing strategies for power and posture control in bionic cross-medium vehicles. Wang et al. [25] conducted numerical simulations on the plunge-diving behavior of gannets, elucidating the influence of the drop height and water entry angle on impact acceleration and revealing the diving strategies of gannets. Hou et al. [26] used CFD methods to simulate the entire process of a flying squid launching from the water into the air, confirming through quantitative analysis of kinematic parameters that jet propulsion strategies can generate greater time-averaged thrust. Deng et al. [27] used CFD methods to numerically study the aerodynamic performance of three flying fish species, not only validating the longitudinal aerodynamic stability of flying fish models but also predicting their gliding range through dynamic models. In summary, many fluid flow problems involving bionics have been solved using CFD methods. This can provide efficient numerical experiments under the condition that the theoretical model is correctly established. Therefore, we used the CFD method to numerically investigate the impact-mitigation mechanisms and dynamic characteristics of swan webbed-foot gliding entry.

In this study, we extracted the key physical characteristics of the swan body and webbed feet using the reverse modeling method, and we designed a simplified three-dimensional bionic webbed-foot model. This study utilized Reynolds-Averaged Navier–Stokes (RANS) equations and the Volume of Fluid (VOF) method to simulate the entire free-motion process of a swan’s webbed foot from impact above the water surface to gliding stop, combined with global motion mesh (GMM) technology to update the six-degrees-of-freedom motion process of the flow-field mesh. Furthermore, we explored the influence of different water-entry velocities, velocity angles, and pitch angles on the gliding water-entry motion of swans, providing bionic inspiration for the design of cross-medium vehicles. This paper offers new perspectives on the air–water cross-medium motion of amphibians, holding significant theoretical and practical importance.

## 2. Three-Dimensional Model and Basic Assumptions of Swan’s Water Entry

### 2.1. Basic Considerations and Assumptions

**Figure 1:**
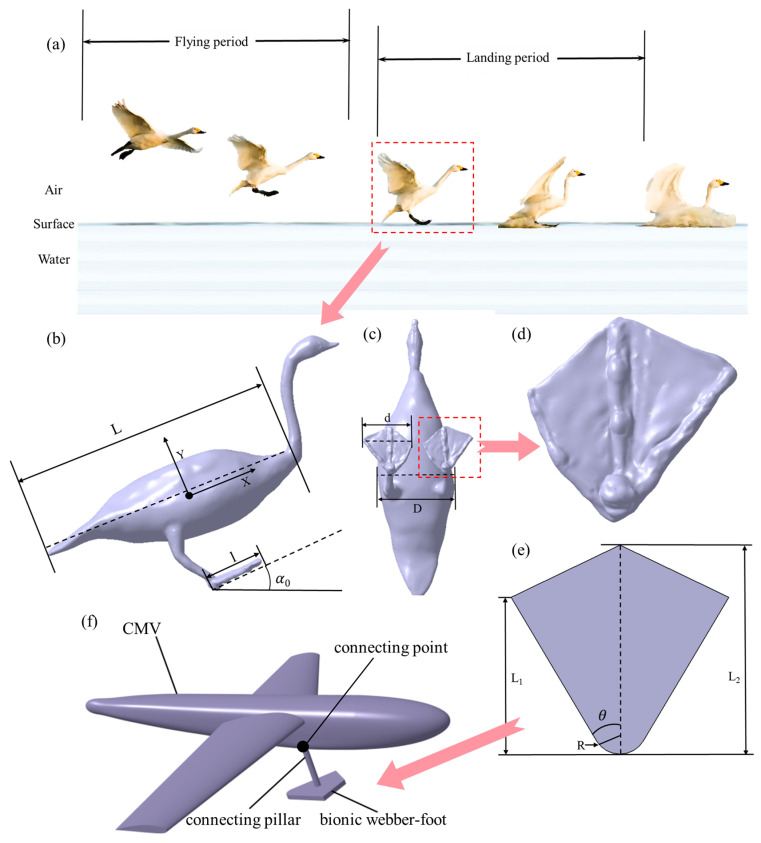
The two periods of a swan’s water-entry process and the 3D swan model: (**a**) two periods of a swan’s water-entry process; (**b**) lateral view of swan body; (**c**) ventral view of swan body; (**d**) bottom configuration of swan’s webbed foot; (**e**) parametric configuration of bio-inspired swan webbed foot; (**f**) schematic diagram of CMV.

Currently, research on the motion processes of large waterfowls primarily focuses on water surface takeoff [28] and migratory flight [29,30], while quantitative studies on the impact loads and motion trajectories during the water-entry process remain scarce. However, among the three phases of swan motion—water exit, flight, and water entry—the water-entry process is the most intricate and perilous, requiring the optimal body posture and entry velocity. In particular, the millisecond-level collision with the water, a transient dynamic process, may lead to bodily injury or motion instability, which underscores the significance of this study. The water-entry process of swans can be divided into two periods, as illustrated in Figure 1a. Initially, during the flying period, the swan adjusts its wing configuration and body pitch angle to simultaneously reduce both the flight velocity and altitude. As it approaches the water surface, the webbed feet extend from the tail to beneath the abdomen in preparation for water entry. Subsequently, during the landing period, the webbed feet make initial contact with the water surface, utilizing hydrodynamic resistance to initiate deceleration and stabilize the body orientation. The gliding motion effectively distributes the body’s momentum over an extended area, thereby reducing impact loading per unit water volume. With an increasing gliding distance, the swan gradually decelerates until there is complete cessation of motion, achieving stable flotation through coordinated adjustments of wing and foot positioning.

Video S1 reveals that although the swan’s water-entry process exhibits dynamic characteristics, the adjustment amplitudes of its wings and body posture remain relatively minor. As a result, the impact loads during water entry mainly originate from the impact forces generated by the interaction of the underside of the webbed feet with the water, while the rest of the swan’s body has almost no contact with the water surface. Considering that the gliding velocity of swans typically remains at relatively low levels (<15 m/s), the influence of aerodynamic forces is comparatively limited. Therefore, instead of considering the aerodynamic properties of the swan’s body and wings, we focus on analyzing the six-degrees-of-freedom motion process and the hydrodynamic action mechanism when the webbed feet enter the water, aiming at elucidating the basic mechanical properties of the swan when gliding into the water. Another assumption is that the mass is uniformly distributed throughout the whole body.

### 2.2. Three-Dimensional Modeling of the Bionic Webbed Foot and the Coordinate System’s Definition

The reverse modeling method was employed to obtain the geometric and physical characteristic parameters of the swan. Firstly, taking the adult swan as the research object, we utilized a 3D scanner to scan its key parts and obtain the surface point cloud data. Subsequently, the point cloud data were simplified, encapsulated, and spliced by Geomagic Wrap 2015 software. Finally, we constructed the 3D model of the swan, as shown in Figure 1b–d, and the definitions of the main parameters are given in Table 1. In order to simplify the CFD calculation, we designed a parameterized 3D bionic webbed foot based on the actual dimensions of the swan’s webbed foot, as shown in Figure 1e. This model requires only five parameters to define its basic configuration: root blunt radius R = 20 mm, root angle θ = 60°, root length L1 = 120 mm, total length L2 = 160 mm, and thickness t = 15 mm. The bionic webbed foot designed in this paper can be installed at the bottom of the CMV by connecting the pillar, as shown in Figure 1f. This pillar can be rotated around the connection point by means of a servo motor. When CMVs cruise in the air or water, the connecting pillar and the bionic webbed foot will fold upwards to reduce the cruise resistance. Before the CMV enters the water, the connecting pillars and the bionic webbed foot will rotate downward to enter the water at the optimal water-contacting angle. The numerical simulation analysis of the bionic webbed foot presented in the following text is based on the application to the CMV.

**Figure 2:**
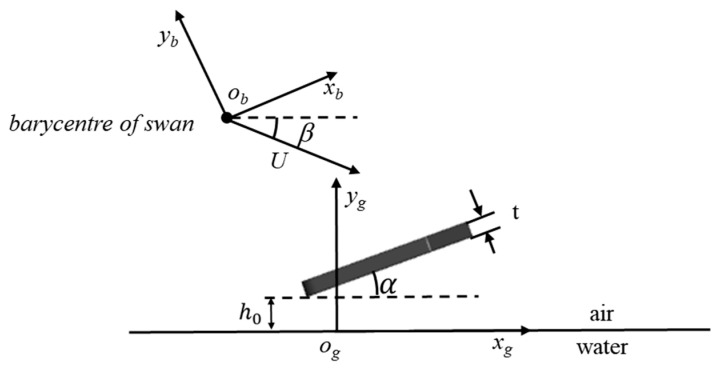
Coordinate systems for swan’s webbed foot.

The numerical simulation study of the webbed foot’s gliding water entry is based on two coordinate systems: the ground and body coordinate systems, as shown in Figure 2. The o_g_-x_g_y_g_z_g_ coordinate system is the ground coordinate system, where the origin o_g_ is the initial contact point between the webbed foot and the free liquid surface. The o_g_y_g_ axis is perpendicular to the initial static water surface, the o_g_-x_g_z_g_ plane lies on the initial static horizontal plane, and the o_g_-x_g_y_g_ plane is the longitudinal plane. The o_b_-x_b_y_b_z_b_ coordinate system is the body coordinate system, established at the center of mass of the swan’s body, where the o_b_-x_b_ axis is parallel to the bottom surface of the webbed foot, the o_g_z_g_ axis is parallel to the o_b_z_b_ axis pointing outward, and the o_b_-x_b_y_b_ plane is perpendicular to the static water surface. The angle of attack *α* of the webbed foot is the angle between the webbed foot and the horizontal plane, and the water-entry velocity angle *β* is the angle between the velocity vector U acting on the barycenter of the swan and the o_g_x_g_ axis. The transformation matrix C*_T_* from the o_g_-x_g_y_g_z_g_ coordinate system to the o-x_b_y_b_z_b_ coordinate system is given by

(1) $$C_{T}=\left(\begin{array}{lll} \cos\alpha & -\sin\alpha & 0\\ \sin\alpha & \cos\alpha & 0\\ \;0 & \;0 & 1 \end{array}\right)$$

## 3. Numerical Calculation Method

We focused on the gliding motion of the webbed foot on water surfaces under low-speed conditions in this study. We assumed that both water and air are incompressible fluids and that there is no heat exchange during the motion. The influence of gravitational acceleration was considered, while surface tension was neglected. In this study, the three-dimensional double-precision solver was used for the simulation calculation in Fluent 2020 R2 software. The numerical calculations employed the unsteady Reynolds-Averaged Navier–Stokes (RANS) equations as the governing equations for fluid flow. The turbulence model selected was the *k-ε* model with standard wall functions, and the multiphase flow field was modeled using the Volume of Fluid (VOF) method. The gliding motion was solved using the six-degrees-of-freedom (6DOF) motion equations. The pressure and velocity fields were coupled using the coupling algorithm, time discretization adopted an implicit scheme, and spatial discretization used a second-order upwind scheme. This computational format was applied to all cases in this study.

### 3.1. Governing Equations and Turbulence Model

The gliding water-entry process of swans involves the interaction of three phases: air, water, and the webbed foot. During this period, the gas–liquid mixture at the air–water interface forms a multiphase flow, and thus the VOF multiphase flow model was employed to capture the gas–liquid interface. In the VOF model, the motion of the air–water interface is tracked through the distribution of *α_a_*, where *α_a_* = 0 represents the liquid phase and *α_a_* = 1 represents the gas phase. By solving the continuity equation and momentum equation, the phase distribution and interface shape are captured [31]. For multiphase flows without interphase mass transfer, the RANS equations can be expressed as

(2) $$\frac{\partial \rho_{m}}{\partial t}+\frac{\partial (\rho_{m}u_{j})}{\partial x_{j}}=0$$

(3) $$\frac{\partial (\rho_{m}u_{i})}{\partial t}+\frac{\partial (\rho_{m}u_{i}u_{j})}{\partial x_{j}}=-\frac{\partial \rho }{\partial x_{i}}+\frac{\partial }{\partial x_{j}}\left[\left(\mu_{m}+\mu_{t})(\frac{\partial u_{i}}{\partial x_{j}}+\frac{\partial u_{j}}{\partial x_{i}}\right)\right]$$

(4) $$\rho_{m}=\rho_{a}\alpha_{a}+\rho_{w}\alpha_{w}$$

(5) $$\mu_{m}=\mu_{a}\alpha_{a}+\mu_{w}\alpha_{w}$$

In the above equations, $u_{i}$ and $u_{j}$ are the Cartesian components of the time-averaged velocity (*i*, *j* = 1, 2, 3); $\rho_{w}$ and $\rho_{a}$, respectively, represent the density of water and air; and $\mu_{w}$ and $\mu_{a}$ represent the dynamic viscosity coefficient of water and air, $\alpha_{a}+\alpha_{w}=1$. $\rho_{m}$ and $\mu_{m}$, respectively, represent the density of the mixed medium and the dynamic viscosity coefficient of the mixed medium. $\mu_{t}$ is the turbulent viscosity coefficient, which can be calculated using the standard k−ε model [32] as follows:

(6) $$\frac{\partial (\rho_{m}k)}{\partial t}+\frac{\partial (\rho_{m}ku_{i})}{\partial x_{i}}=\frac{\partial }{\partial x_{j}}\left[(\mu_{m}+\frac{\mu_{t}}{\sigma_{k}})\frac{\partial k}{\partial x_{j}}\right]+G_{k}+G_{b}-\rho \varepsilon -Y_{M}$$

(7) $$\frac{\partial (\rho_{m}\varepsilon )}{\partial t}+\frac{\partial (\rho_{m}\varepsilon u_{j})}{\partial x_{i}}=\frac{\partial }{\partial x_{j}}\left[(\mu_{m}+\frac{\mu_{t}}{\sigma_{k}})\frac{\partial k}{\partial x_{j}}\right]+C_{1\varepsilon }\frac{\varepsilon }{k}G_{k}-C_{2\varepsilon }\rho \frac{\varepsilon^{2}}{k}$$

where $G_{k}$, $G_{b}$, and $Y_{M}$ can be derived and calculated by references [33,34]. Therefore, the turbulent viscosity coefficient $\mu_{t}$ can be calculated by combining *k* and ε:

(8) $$\mu_{t}=\frac{C_{\mu }\rho_{m}k^{2}}{\varepsilon }$$

In Formulas (7) and (8), $C_{1\varepsilon }$, $C_{2\varepsilon }$, and $C_{\mu }$ are constants.

### 3.2. Global Motion Mesh Technology and 6-Degrees-of-Freedom Motion Model

This study employed global motion mesh (GMM) technology to simulate the gliding water-entry process of the bionic webbed foot. The principle involves the computational domain moving as a rigid body along with the swan’s webbed foot. During computation, an inertial coordinate system is used. At each time step, the CFD software converts the obtained hydrodynamic forces and moments into translational and rotational motions of the body, further calculating the body’s motion posture. The grid is continuously updated for iterative solving, ultimately achieving the coupling of the flow field and motion trajectory. The unsteady volume-fraction boundary condition ensures that the free water surface remains horizontal in the inertial coordinate system as the computational domain moves. Specifically, in the ground coordinate system, the volume fraction in the boundary grid of the computational domain is set to 0.5 at the air–water interface, 0 above the water surface, and 1 below the water surface. The advantage of the overset grid motion method is that it eliminates the need for any grid reconstruction or deformation techniques, ensuring grid quality throughout the computational domain. This improves numerical accuracy (especially in capturing the free water surface) and computational stability. Additionally, since it is unnecessary to model the entire water volume traversed during the gliding water-entry process, the number of grids is significantly reduced, greatly saving computational costs [35].

The 6DOF model solves the translational and rotational equations to determine the position of the center of mass and the orientation of the object. The translational equations are solved in the inertial coordinate system:

(9) $$\overset{\cdot }{v_{g}}=\frac{1}{m}\sum f_{g}$$

where *m* is the mass of the swan; $v_{g}$ is the translational speed of the center of gravity; and $f_{g}$ is the sum of the force and gravity of the swan, with the subscript *g* denoting the ground coordinate system.

The rotation equation is solved in the body coordinate system:

(10) $$\overset{\cdot }{\omega_{g}}=K^{-1}(\sum M_{b}-\omega_{b}\times K\omega_{b})$$

In the above equation, K represents the inertia tensor; the subscript *g* represents the ground coordinate system; the subscript *b* represents the body coordinate system; ω represents the angular velocity of the body around the center of gravity; and $M_{b}$ is the moment acting on the swan about its center of mass.

### 3.3. Computational Domain and Boundary Conditions

**Figure 3:**
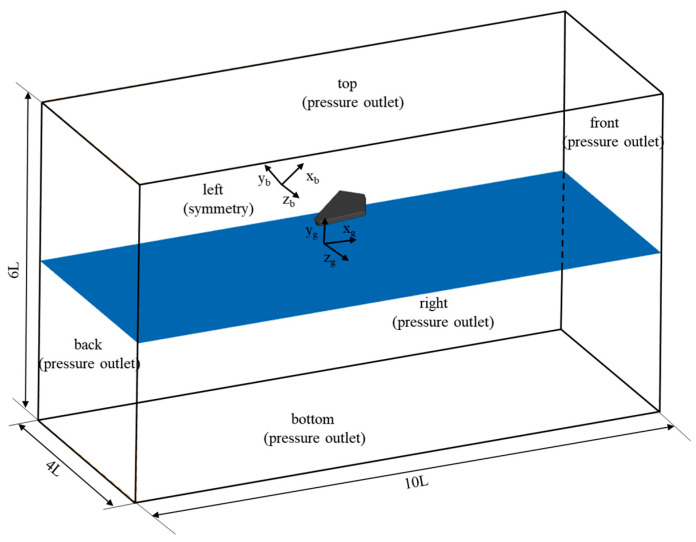
Schematic diagram of the computational domain and boundary conditions.

In the numerical simulation, three types of boundary conditions are used to define the computational domain: pressure outlet, symmetry plane, and wall boundaries. As shown in Figure 3, the computational domain has a length of 10 L along the horizontal direction (o_g_x_g_ axis), a height of 6 L along the vertical direction (o_g_y_g_ axis), where the height of the water is 3 L, and a width of 4 L along the lateral direction (o_g_z_g_ axis). The top and bottom boundaries in the vertical direction, the front and rear boundaries in the horizontal direction, and the right boundary in the lateral direction are all set as pressure outlet boundaries.

The initial pressure in the computational domain was set as follows: before the simulation began, the pressure in the air region (above the o_g_x_g_y_g_ plane) was maintained at the standard atmospheric pressure. In the water region below the o_g_x_g_y_g_ plane, the pressure was determined by the hydrostatic pressure formula p=ρgh, where *h* is the depth from the water surface. At each computational time step, the pressure at the pressure outlet boundaries was updated based on the height in the ground coordinate system, following the same method as the initial pressure setting. The left boundary in the lateral direction was set as a symmetry plane boundary condition to simulate the symmetrical characteristics of the swan’s body. Moreover, the distance from the left boundary to the webbed foot was equal to half of the actual distance between the two feet. The swan’s webbed foot was set as an adiabatic, no-slip wall boundary to accurately reflect its interaction with the fluid.

### 3.4. Mesh Independence Verification

**Figure 4:**
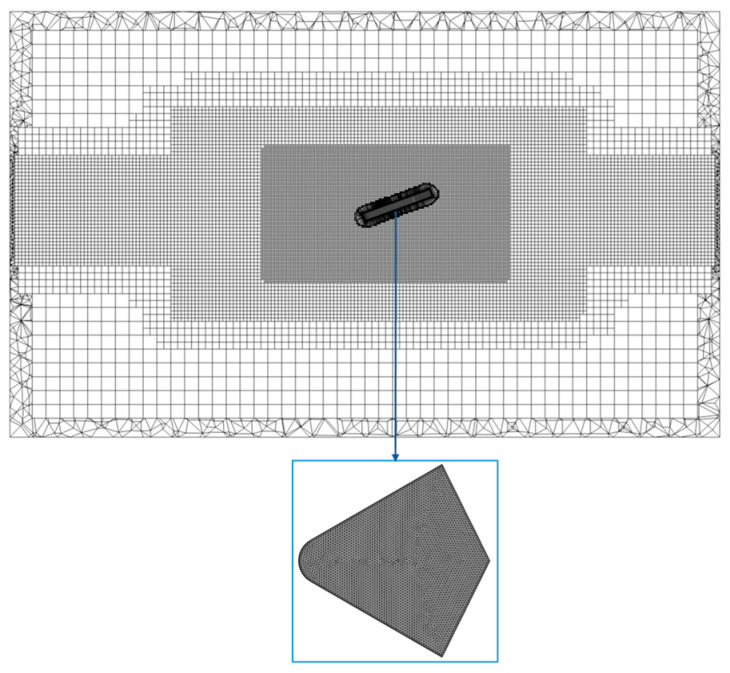
Computational domain mesh configuration.

In this section, we presented the mesh independence study to determine the appropriate mesh size for modeling the water-entry process of the swan’s webbed foot. The computational domain was discretized by using the Hexcore hybrid meshing method in Fluent Meshing 2020 R2 software. This method generates structured hexahedral grids in the core area of the flow field to ensure high-resolution discretization of critical flow areas, while automatically filling complex curved surfaces and transitional regions with unstructured tetrahedral grids. Particular attention is given to the areas near the webbed foot, where a refined mesh zone is implemented. The middle grid is shown in Figure 4. The refined zone mesh size of the medium mesh was set to 4 mm, while the far-field region mesh size of the medium mesh was set to 32 mm. The surface mesh size of the webbed foot was set at 0.6 mm, with the y+ value falling within the effective range of the standard wall function [36].

**Figure 5:**
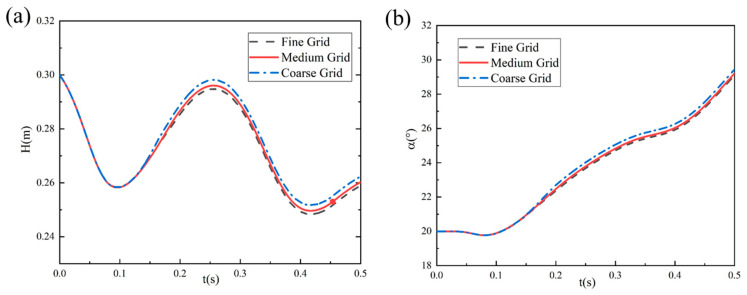
Results of mesh convergence analysis: (**a**) temporal variation in swan’s centroid height; (**b**) temporal variation in webbed foot’s angle of attack.

Based on the mesh model shown above, three sets of progressively refined computational grids were established to verify mesh independence. The case of a 6 m/s entry velocity, 20° initial pitch angle, and 3° entry velocity inclination angle was chosen to perform this grid sensitivity verification. Three distinct mesh configurations were generated by adjusting the refinement zones and element sizes: coarse mesh (1.4078 million elements), medium mesh (2.8066 million elements), and fine mesh (5.6821 million elements). The refined zone mesh sizes of the coarse mesh, medium mesh, and fine mesh were respectively 8 mm, 4 mm, and 2 mm, while the far-field region mesh size of all three types of grids remain at 32 mm. Figure 5 presents the variation curves of the swan’s centroid height *H* and angle of attack *α* versus time t obtained from numerical simulations using these three grid resolutions. The results demonstrate that the computational outcomes between the medium and fine grids show minimal differences, with their trajectory evolution curves nearly coinciding, while the coarse grid exhibits noticeable deviations in motion trajectory. Considering both computational efficiency and accuracy, we adopted the medium grid resolution for numerical simulations of the bionic webbed foot’s water-entry motion in this paper. This selection ensures computational precision while significantly reducing computational costs, making it suitable for large-scale numerical simulations.

### 3.5. Validation of Numerical Methods

To verify the accuracy of the numerical method, this study conducts a numerical simulation of the water surface skipping process of a disk based on the experimental conditions described in reference [37]. The experiment used an aluminum disk with a thickness *D* = 2.75 mm, radius *R* = 25 mm, and mass *M* = 0.014 kg. A half-model simulation is employed, with a surface grid size of 0.6 mm and a total of 1.24 million grids. The model is positioned at the center of a semi-cubic computational domain, with the model’s symmetry plane coinciding with the symmetry plane of the computational domain. The disk plane is parallel to the top and bottom boundaries of the computational domain. The boundaries of the computational domain are located at a distance of five times the disk diameter from the disk’s center of mass. The symmetry plane uses a symmetry boundary condition, and the outer field boundaries are set as pressure outlets. The computational conditions include a water-entry velocity *v* = 3.5 m/s, an angle of attack *α* = 35°, and a velocity angle *β* = 20°.

**Figure 6:**
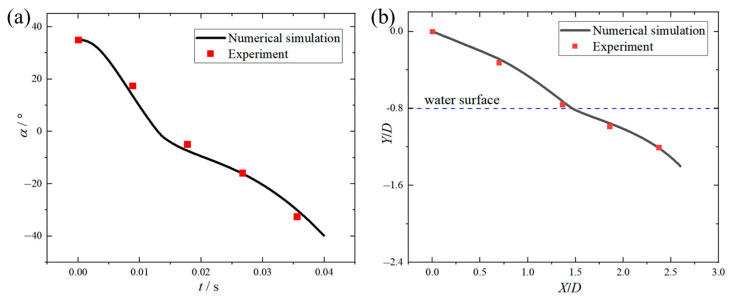
Comparison of numerical simulation and experimental results for motion parameters during disk skipping near water surface: (**a**) variation curve of pitch angle versus water contact time; (**b**) variation curve of disk head displacement versus time.

Figure 6 shows the temporal variations in both pitch angle and head displacement during the disk gliding motion, with comparative validation against experimental data [37]. Pearson correlation analysis was conducted on the numerical results and the experimental results. The correlation coefficient of the pitch angle was 0.9972, with a *p*-value of 0.00282; the correlation coefficient of the head displacement was 0.9992, with a *p*-value of 0.0008. The analysis results indicated that the numerical simulation results were significantly positively correlated with the experimental results, verifying the predictive capability of the present computational methodology for water-surface-gliding phenomena. Based on this validation, the identical surface mesh resolution (0.6 mm) was employed in subsequent simulations.

## 4. Results Analysis and Discussion

### 4.1. Motion Characteristics and Flow-Field Analysis of Bionic Swan Webbed Foot During Typical Water-Entry Process

**Figure 7:**
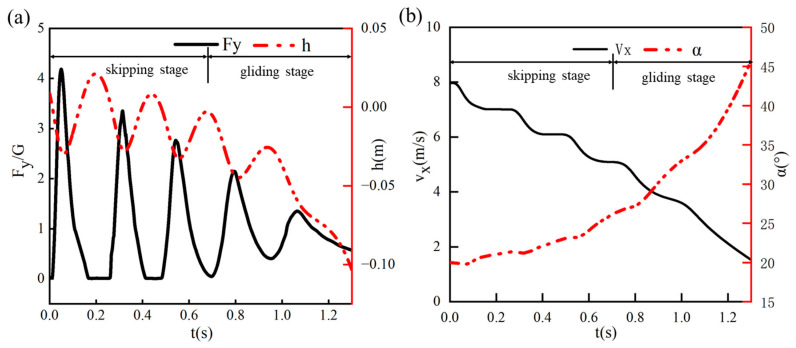
Temporal variations in kinematic parameters and hydrodynamic loads for the bio-inspired webbed foot: (**a**) vertical displacement and vertical impact load; (**b**) horizontal velocity and pitch angle.

To investigate the motion characteristics and flow-field patterns during the water-entry process of the bionic swan webbed foot, a typical working condition was selected with an entry velocity U_0_ = 8 m/s, initial pitch angle α_0_ = 20°, and entry velocity inclination β_0_ = 5°. The entire process from water contact to the end of hydroplaning was simulated. Figure 7 illustrates the temporal variations in vertical displacement, vertical impact overload F_y_/G (where F_y_ represents the vertical impact load and G denotes the swan’s gravity), horizontal velocity, and pitch angle during the hydroplaning process of the bionic webbed foot.

We defined a complete skip-gliding cycle as the hydrodynamic phenomenon where the bionic webbed foot impacts the water surface and subsequently skips out again. Through systematic analysis of kinematic parameter curves, the water-entry process of the bionic webbed foot can be classified into two primary phases: the skip-gliding phase and the surface-gliding phase. During the initial 0.7 s skip-gliding phase, the swan’s vertical center of mass demonstrates characteristic quasi-sinusoidal oscillations. Significantly, the minimum center-of-mass position in each skip cycle correlates precisely with the instant of maximum vertical impact overload. As the skip-gliding process progresses, the system exhibits progressive kinetic energy dissipation, evidenced by two distinct phenomena: A monotonic decrease in peak impact load and progressive reduction in inter-peak duration. Following three complete skip-gliding cycles, the system transitions into the surface-gliding phase, where the impact load fluctuations on the webbed foot attenuate significantly to levels insufficient for water-exit propulsion, accompanied by a continuous decrease in center-of-mass height, accelerated kinetic energy dissipation rate, and rapid reduction in horizontal velocity below 2 m/s, ultimately achieving smooth water-surface landing through progressive hydrodynamic stabilization and efficient energy absorption mechanisms.

**Figure 8:**
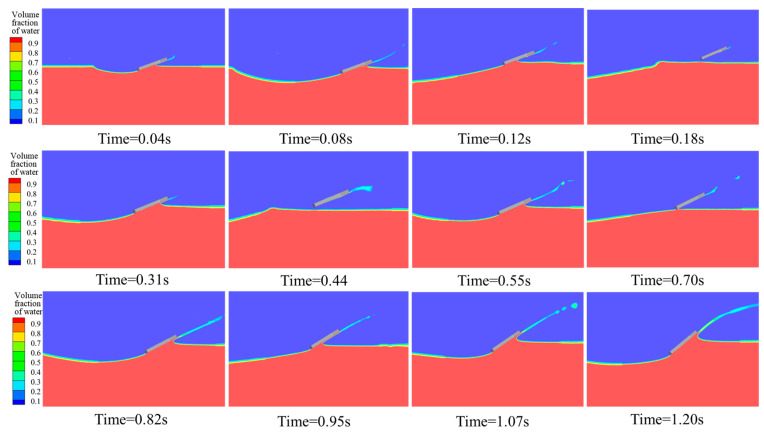
Contour plots of free surface morphology during the bio-inspired webbed foot’s gliding water-entry process.

Figure 8 further illustrates the positional changes in the webbed foot relative to the free surface throughout the water-entry process, where the gray rectangles represent the webbed-foot. At t = 0.01 s, the bionic webbed foot makes initial contact with the water surface, engaging in momentum exchange with the surrounding fluid and generating a substantial cavity along with surface waves. Under the influence of impact loads, the bionic webbed foot skips out of the water and hydroplanes near the surface for a certain distance. The second and third skipping events occur at t = 0.33 s and t = 0.55 s, respectively. Compared to the first skip, these subsequent events exhibit significantly reduced bounce heights and diminished free surface deformation amplitudes. After 0.7 s, the system transitions into the surface-gliding phase where the bio-inspired webbed foot exhibits a continuous increase in pitch angle due to the nose-up moment, significantly enlarging its horizontal contact area with water and thereby enhancing velocity attenuation. The gliding mechanism enables momentum distribution across a broader water volume, substantially reducing the impact load per unit area. During this phase, cavity expansion occurs progressively while the separating splash flow forms distinct water jets at the leading edge that subsequently collapse back to the free surface, visually demonstrating the system’s vigorous energy dissipation process.

**Figure 9:**
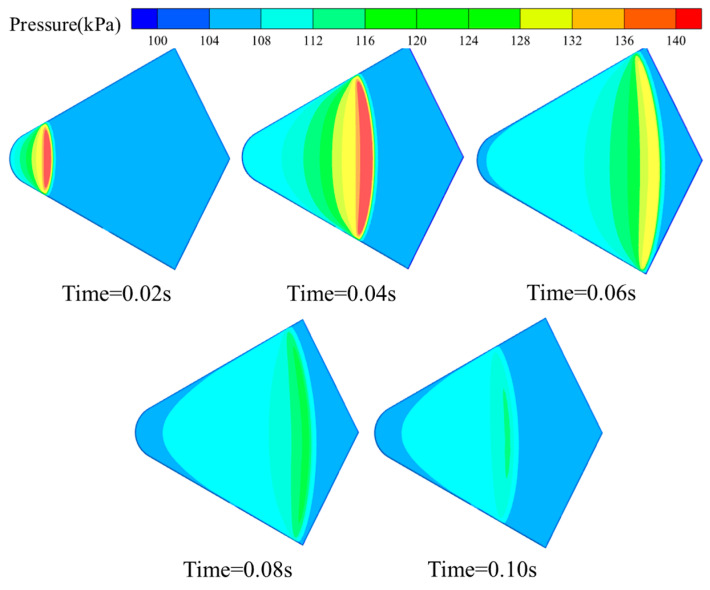
Contour plot of bottom surface pressure distribution during initial water impact of bionic webbed foot.

To further analyze the load evolution during the bionic webbed foot’s gliding water-entry process, we presented the pressure distribution nephograms on the bottom surface during the initial glide phase, shown in Figure 9. At the moment of impact into the water, a local high-pressure area is formed at the tail of the bottom surface of the bionic webbed foot, and the pressure is symmetrically distributed along the center axis of the bottom surface. With the increase in water depth, the high-pressure area gradually moves to the head and extends to the whole bottom surface of the webbed foot, and the pressure peak is concentrated near the root of the jet and causes the pressure to rise in a large area of water below the webbed foot. As the bionic webbed foot gradually slipped out of the water, the high pressure distribution on the bottom surface of the webbed foot gradually dissipated from the front to the back of the webbed foot, and the pressure peak decreased significantly. The spatial and temporal evolution of the pressure distribution reveals the mechanical mechanism of the bionic webbed foot during the water-entry process: at the initial stage of water entry, the impact load plays a dominant role, subjecting the webbed foot to a high peak load and forming a localized high-pressure zone; with the continuation of the gliding movement, the resistance of the water body to the webbed foot gradually becomes the main influencing factor, which manifests as a significant decrease in the peak pressure in the touching area. This finding clarifies the mechanical nature of the gliding load-reduction behavior of the swan’s webbed foot: the gradual dissipation of kinetic energy is achieved through the progressive gliding resistance of the water body on the webbed foot, which avoids the high impact load due to the instantaneous release of energy during the direct impact with the water.

### 4.2. Influence of Initial Motion Parameters on Bionic Webbed Foot’s Motion Characteristics

The gliding water-entry motion of the bionic webbed foot is influenced by multiple factors, including structural parameters, entry velocity (U_0_), velocity angle (β_0_), and pitch angle (α_0_). For the specific bionic webbed-foot configuration studied in this work, we focus on investigating how these three initial motion parameters affect the gliding water-entry dynamics and load-mitigation performance.

#### 4.2.1. Effect of Initial Velocity

**Figure 10:**
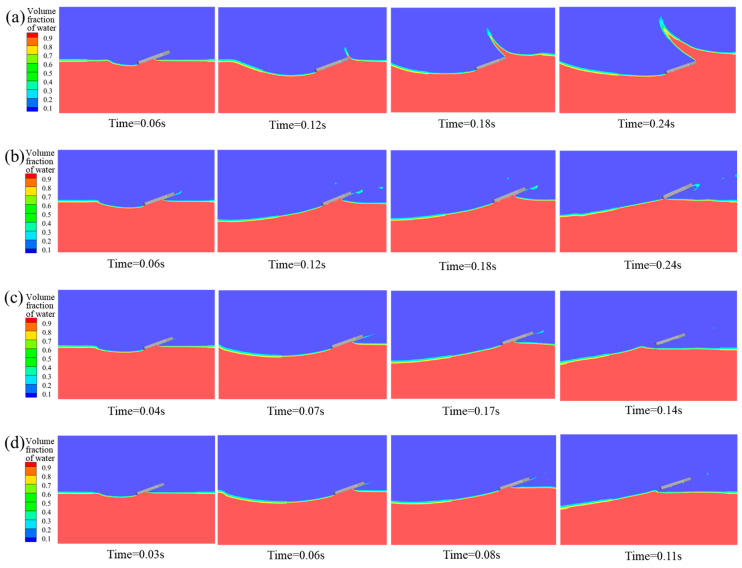
Free surface morphology during water entry at different initial velocities: (**a**) v_0_ = 4 m/s; (**b**) v_0_ = 6 m/s; (**c**) v_0_ = 10 m/s; (**d**) v_0_ = 12 m/s.

To investigate the influence mechanism of entry velocity on the water entry motion of bionic webbed foot, numerical simulations were conducted for cases with an initial pitch angle α_0_ = 20°, entry velocity inclination β_0_ = 5°, and initial entry velocities U_0_ of 4 m/s, 6 m/s, 8 m/s, 10 m/s, and 12 m/s. Figure 10 presents the free surface contours induced by the initial water-entry process of the bionic webbed foot at different initial entry velocities. When the entry velocity is 4 m/s, the impact load on the bionic webbed foot is insufficient to overcome gravity for water exit, resulting in the leading edge submerging below the free surface after a short hydroplaning distance. During this process, the hydroplaning descent generates a substantial cavity with noticeable liquid separation at the leading edge. At 6 m/s entry velocity, the hydrodynamic force remains inadequate for complete water exit, representing a critical skipping state where the webbed foot hydroplanes precisely at the water surface after skipping. For entry velocities ranging from 8 m/s to 12 m/s, the bionic webbed foot successfully skips out of the water after impact, creating large cavities at the surface depression. As the initial entry velocity increases, the duration of the initial water-entry process shortens, with a reduced entry depth and cavity volume. This phenomenon occurs because higher initial entry velocities significantly increase the impact load on the bionic webbed foot, accelerating the water exit velocity and consequently reducing the hydroplaning duration and energy dissipation rate. These results demonstrate that the initial entry velocity substantially influences the motion characteristics of the bionic webbed foot, particularly playing a crucial role in skipping behavior and cavity formation.

Table 2 presents the skipping frequency and skipping distance of the bionic webbed foot under different water-entry velocities. The table reveals that both the skipping frequency and hydroplaning distance significantly increase with higher entry velocities. Specifically, when the entry velocity increases from 8 m/s to 10 m/s, the skipping frequency rises from three to five times (a 66.7% increase), and the stable skipping distance extends from 4.49 m to 8.81 m (a 96.2% increase). When the entry velocity further increases from 10 m/s to 12 m/s, the skipping frequency increased from five to eight times (a 60.0% increase), and the stable skipping distance extends from 8.81 m to 15.37 m (a 74.5% increase). Pearson correlation analysis was conducted on the skipping frequency and the skipping distance under different water-entry velocities. The correlation coefficient was 0.9999, and the *p*-value was 0.00213. This indicates that as the water-entry velocity increases, the increase in the skipping frequency is significantly positively correlated with the increase in the skipping distance. The fundamental mechanism arises because an increased entry velocity substantially elevates the initial kinetic energy of the bionic webbed foot, necessitating both a greater skipping frequency and extended gliding distance for complete energy dissipation. Only when repeated stable skips reduce the kinetic energy below a critical threshold can the webbed foot transition to gradual deceleration through surface gliding, ultimately achieving stable water surface arrest.

**Figure 11:**
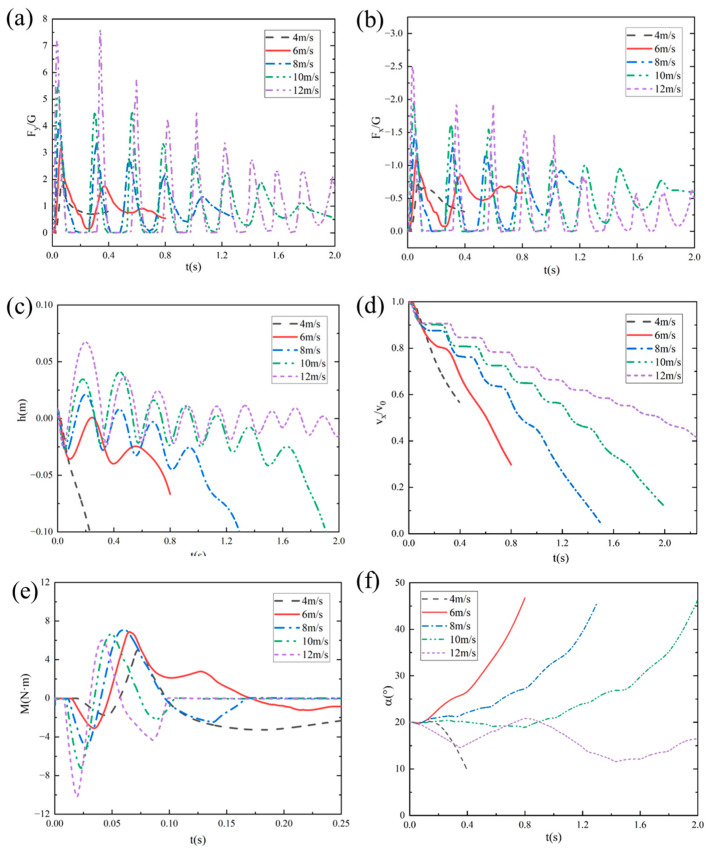
Temporal variation curves of motion characteristic parameters under different water-entry velocity conditions: (**a**) vertical impact overload; (**b**) horizontal impact load; (**c**) distance from the bottom of the webbed foot to the water surface; (**d**) dimensionless horizontal velocity; (**e**) pitching moment; (**f**) pitch angle.

Figure 11 shows the temporal variation curves of vertical impact overload (F_y_/G), horizontal impact overload (F_x_/G), the distance from the bottom of the webbed foot to the water surface (h), the dimensionless horizontal velocity (v_x_/v_0_), the pitching moment (M), and the pitch angle (α) for the bionic webbed foot at different entry velocities. When the entry velocity is 4 m/s, the bionic webbed foot exhibits continuous center-of-gravity descent after water impact, eventually submerging completely. During this process, hydrodynamic forces generate a relatively stable nose-down moment, leading to a rapid decrease in pitch angle. When the entry velocity is 6 m/s, the bionic webbed foot glides on the water surface post-impact, with the center of gravity oscillating vertically without complete water exit. As kinetic energy dissipates, the webbed foot gradually submerges. When the entry velocities exceed 8 m/s, the bionic webbed foot undergoes multiple skipping motions after impact, followed by stable gliding entry. With increasing entry velocity, the initial water contact duration shortens, the peak impact load during first contact increases, the maximum skipping height rises, and the number of skipping cycles grows. Throughout the skipping process, peak loads exhibit a decreasing trend with each subsequent impact, accompanied by kinetic energy dissipation. Higher initial entry velocities correspond to lower kinetic energy dissipation rates during skipping and slower horizontal velocity decay.

The pitching moment demonstrates a characteristic pattern: an initial negative peak followed by a positive peak, eventually stabilizing. This behavior can be attributed to the following mechanisms: During initial water contact, the load acting on the bottom surface generates a nose-down moment from horizontal forces that exceeds the nose-up moment from vertical forces. As water entry depth increases, the resultant force position shifts toward the leading edge, shortening the vertical force arm and lengthening the horizontal force arm, causing the pitching moment to transition from nose-down to nose-up. During an upward skipping motion, the reduced impact load on the bionic webbed foot leads to stabilization of the pitching moment. When the entry velocity is 6 m/s, the bionic webbed foot experiences a relatively small nose-down moment during water entry, with the nose-up moment dominating, resulting in a rapid pitch angle increase. Conversely, at higher entry velocities, the nose-down moment gradually becomes dominant, causing the bionic webbed foot to exhibit an initial nose-down followed by nose-up motion trend.

#### 4.2.2. Influence Mechanism of Initial Pitch Angle

**Figure 12:**
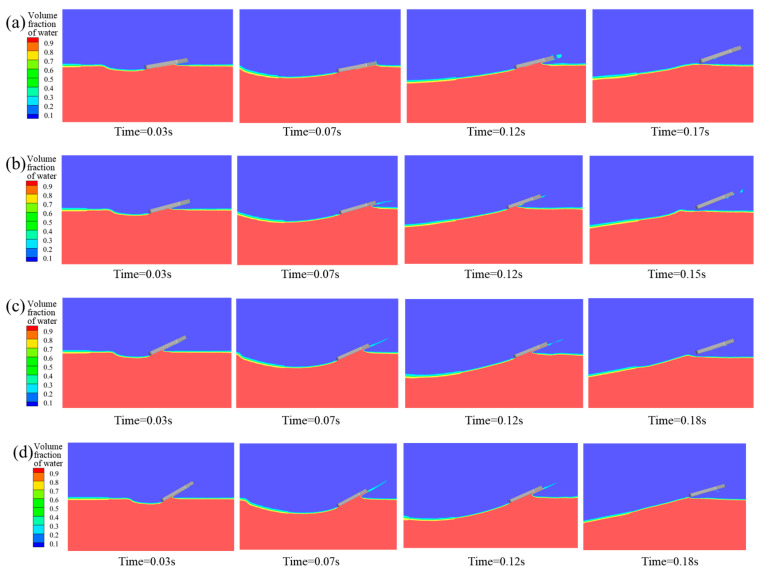
Free surface morphology during water entry at different initial pitch angles: (**a**) α_0_ = 10°; (**b**) α_0_ = 15°; (**c**) α_0_ = 25°; (**d**) α_0_ = 30°.

To study the influence mechanism of the initial pitch angle on the bionic webbed foots’ entry motion in detail, numerical simulation was carried out for the initial entry speed of U_0_ = 8 m/s, entry speed inclination β_0_ = 5°, and initial pitch angles of 10°, 15°, 20°, 25°, and 30°. Figure 12 shows the evolution characteristics of the free liquid surface during the bionic foot fins’ first entry into water at different initial pitch angles. The research shows that when the pitch angle is 10°~30°, the bionic webbed foot can realize the complete sliding movement. Specifically, when the pitch angle is 10°, the head of the webbed foot is in contact with the water surface, causing the water body to flow along the head to the surface, forming a large wet area, and the liquid surface separation phenomenon is weak. With the increase in pitch angle, the water spatter intensity of the front edge of the feet increases significantly, and the void volume of the trailing edge expands. This phenomenon indicates that increasing the pitch angle can increase the contact area between the horizontal direction of the feet and the water body, thus intensifying the energy exchange during the water-entry impact.

**Figure 13:**
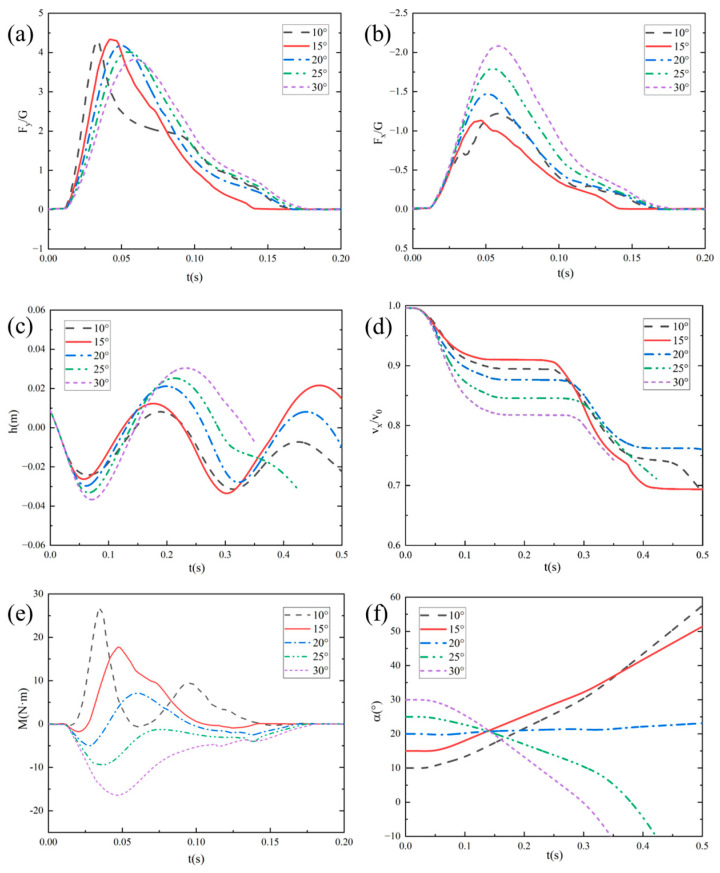
Temporal variation curves of motion characteristic parameters under different water-entry pitch angles: (**a**) vertical impact overload; (**b**) horizontal impact load; (**c**) distance from the bottom of the webbed foot to the water surface; (**d**) dimensionless horizontal velocity; (**e**) pitching moment, (**f**) pitch angle.

Figure 13 presents the temporal variations in vertical impact overload (F_y_/G), horizontal impact overload (F_x_/G), distance from the bottom of the webbed foot to the water surface (h), dimensionless horizontal velocity (v_x_/v_0_), pitching moment (M), and pitch angle (α) for the bionic flipper under different water-entry pitch angles. The results demonstrate that at an initial pitch angle of 10°, partial immersion of the flipper head induced minor fluctuations in both impact load evolution and pitching moment. With increasing initial pitch angles, the load evolution curves stabilized progressively. The vertical impact load peak exhibited a marginal reduction, while the horizontal impact load peak increased substantially with a concurrent enhancement of the kinetic energy dissipation rate.

Under smaller initial pitch angles (10° and 15°), the dominant nose-up moment during initial water entry caused significant increases in angular velocity and pitch angle during webbed-foot skipping, potentially compromising the subsequent stable skipping motion of the vehicle. Conversely, under larger initial pitch angles (25° and 30°), though nose-up moments remained predominant, the reduced angular velocity and pitch angle during skipping might have induced head-first water impact, subsequently leading to motion instability.

#### 4.2.3. Influence of Initial Water-Entry Velocity Angle

**Figure 14:**
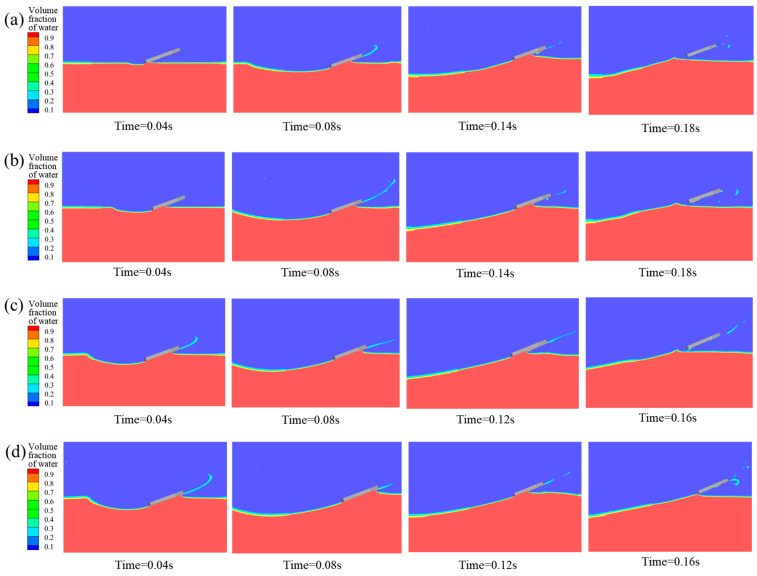
Free surface morphology during water entry at different initial water-entry velocity angles: (**a**) β = 1°; (**b**) β = 3°; (**c**) β = 7°; (**d**) β = 9°.

To investigate the effect of the initial velocity angle on the water-entry motion of the bionic webbed foot, numerical simulations were conducted for cases with an initial entry velocity U_0_ = 8 m/s, pitch angle α_0_ = 20°, and initial velocity angles β = 1°, 3°, 5°, 7°, and 9°. Figure 14 presents the free surface contours induced by the initial water-entry process of the bionic webbed foot at different initial velocity angles. The results demonstrate that the initial stable skipping process exhibits similarities across different velocity angles. Upon water impact, the bionic webbed foot interacts with the water, creating a cavity and causing a water splash near the leading edge. Subsequently, the webbed foot gradually ascends and skips out of the water under the impact load. As the initial velocity angle increases, the water-entry depth significantly increases, the cavity expansion accelerates, and the cavity volume substantially enlarges. This phenomenon occurs because a larger initial velocity angle provides a greater vertical velocity component during water entry, thereby increasing the impact energy on the water and resulting in more intense cavity expansion.

Table 3 presents the skipping frequency and hydroplaning distance of the bionic webbed foot under different water-entry velocity angles. The table reveals that both the skipping frequency and hydroplaning distance decrease with increasing entry velocity angles. Specifically, when the entry velocity angle increases from 1° to 5°, the skipping frequency remains constant at three times, while the stable hydroplaning distance decreases from 4.83 m to 4.49 m (a 7.0% reduction). When the entry velocity angle further increases from 5° to 9°, the skipping frequency decreases from three to two times (a 33.3% reduction), and the stable hydroplaning distance shortens from 4.49 m to 3.10 m (a 31.0% reduction). Pearson correlation analysis was conducted on the skipping frequency and the skipping distance under different water-entry velocity angles. The correlation coefficient was 0.9502, and the *p*-value was 0.01322. This indicates that as the angle of water-entry velocity increases, the decrease in the number of jumps is positively correlated with the shortening of the jump distance. The analysis of this phenomenon is as follows. The vertical impact load formula is

(11) $$F_{y}=C_{y}\rho Av^{2}\sin^{2}\beta$$

where $C_{y}$ is the dimensionless load coefficient, *A* represents the wetted area, and vsinβ is the vertical velocity component. The quadratic relationship between vertical impact load and normal velocity demonstrates that increasing the water entry angle dramatically enhances both the hydrodynamic loading and fluid–structure energy transfer, resulting in more intense expansion of the cavity.

**Figure 15:**
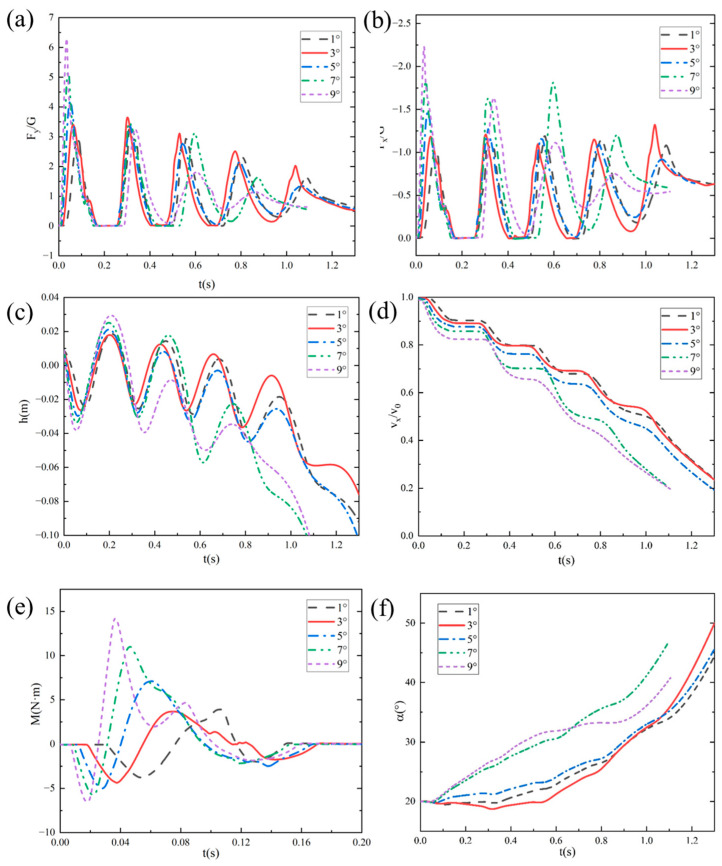
Curve of motion characteristic parameters with time under different water-entry velocity inclination angles: (**a**) vertical impact overload; (**b**) horizontal impact load; (**c**) distance from the bottom of the webbed foot to the water surface; (**d**) dimensionless horizontal velocity; (**e**) pitching moment; (**f**) pitch angle.

Figure 15 shows the change curves of vertical impact overload F_y_/G, horizontal impact overload F_x_/G, the distance from the bottom of webbed foot to the water surface h, dimensionless horizontal velocity v_x_/v_0_, the pitching moment M, and the pitch angle α with time under different entry velocity angles. The analysis results show that the bionic foot fins experience many sliding movements after touching the water, and finally glide into the water through kinetic energy dissipation. With the increase in the dip angle of the water-entry velocity, the duration of the bionic foot’s first contact with water is extended, the vertical and horizontal peak loads increase significantly, and the depth of the first entry and the height of the slide increase correspondingly. According to the hydrodynamic characteristics when sliding into the water, when the dip angle of the sliding speed is 1°, the peak load of the bionic foot fins during sliding is maintained at a low level, and the horizontal speed attenuation is relatively slow. With the increase in the dip angle of the water-entry velocity, the peak value of the vertical impact load during the sliding jump process shows a trend of decreasing successively, and the peak value of the horizontal impact load during the first two water contacts increases obviously, which indicates that the kinetic energy dissipation efficiency has been significantly improved. The mechanism of this phenomenon can be attributed to the following reasons: an increase in the inclination angle of the entry velocity increases the vertical entry velocity of the bionic webbed foot, which enhances the impact of the water on the webbed foot, accelerates the process of kinetic energy dissipation, and increases the vertical impulse and the vertical velocity of the flipper out of the water. In addition, a larger inclination angle of the entry velocity enhances the pitching moment of the bionic webbed foot during the first entry and makes the head-up moment dominant, which leads to a significant increase in the pitching angle during the first slip. This kinematic feature increases the horizontal contact area of the bionic webbed foot during the second water entry, which further enhances the horizontal impact load.

When the water-entry velocity inclination angle was 9°, the head-up moment of the bionic webbed foot’s first water contact rapidly decreased to the trough after reaching the peak, and the double-peak phenomenon appeared. This is due to the fact that the water is deep under this condition, and just the head of the webbed foot is submerged in the water body, which inhibits the continuous growth of the head-up moment, thus generating moment fluctuation characteristics. From the perspective of gliding load reduction, a smaller water-entry inclination can effectively reduce the peak impact load and pitching moment amplitude, which is conducive to maintaining attitude stability during the motion process.

#### 4.2.4. Analysis of Initial Motion Parameters on Dynamic Stability

The analysis of individual initial motion parameters reveals that the entry velocity, inclination angle, and pitch angle collectively influence the stability of the bionic webbed foot during near-surface gliding. Beyond critical thresholds for each parameter, the webbed foot’s dynamic stability undergoes significant transitions, highlighting the necessity of a multi-parametric approach for stability assessment. In this section, we systematically explore the stable operational regime within the three-dimensional parameter space defined by these initial conditions (velocity, inclination angle, and pitch angle) to identify the boundaries of stable motion and characterize transitional behaviors. This comprehensive mapping provides fundamental insights for optimizing bionic webbed-foot performance while avoiding instability during water-entry and gliding phases.

**Figure 16:**
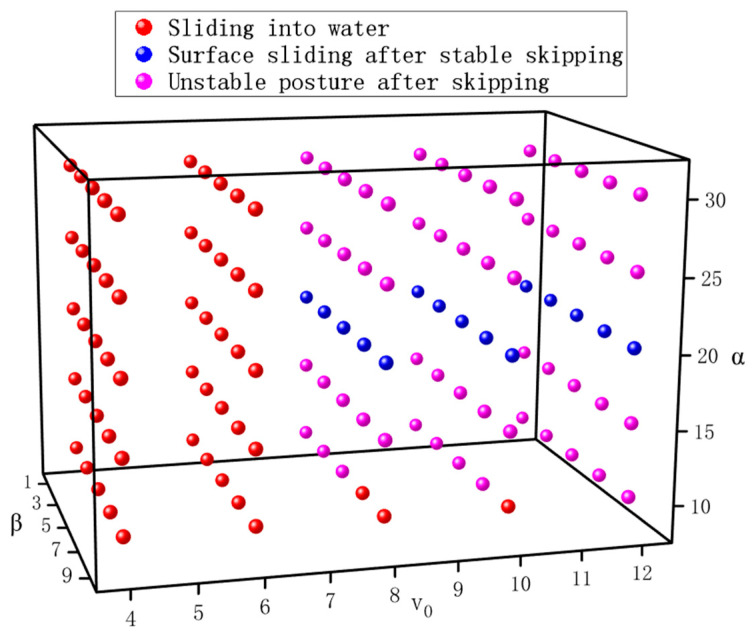
Classification map of motion patterns for the bionic webbed foot.

This study systematically investigated the effects of initial conditions on water-entry dynamics through 125 numerical cases covering five velocities (4–12 m/s), five pitch angles (10–30°), and five inclination angles (1–9°). Through computational analysis, Figure 16 presents a motion-mode classification map of the bionic webbed foot, revealing three distinct motion patterns: (1) direct gliding water entry, (2) stable water skipping followed by gliding entry, and (3) post-skipping instability (where the pitch angle exceeds 60° or becomes negative). This study demonstrates that the entry velocity is the primary determinant of motion patterns. At velocities below 6 m/s, the vertical impact load proves insufficient to sustain water-skipping, resulting in direct surface gliding followed by submersion. The entry pitch angle (α_0_) predominantly governs post-skipping stability—values exceeding 25° or below 15° frequently induce attitude instability, potentially causing high-angular-velocity water impact at either the head or tail section, which may lead to structural damage or even capsizing. Notably, under combined conditions of a large velocity inclination angle (β_0_) and small pitch angle (α_0_), direct gliding water entry persists even at entry velocities up to 10 m/s. This finding suggests that increasing β_0_ promotes a transition from gliding entry to pure impact entry, which compromises effective load mitigation. This systematic investigation of parametric influences on gliding water-entry dynamics provides crucial theoretical foundations and practical guidance for optimizing the bionic webbed foot’s motion parameters.

**Figure 17:**
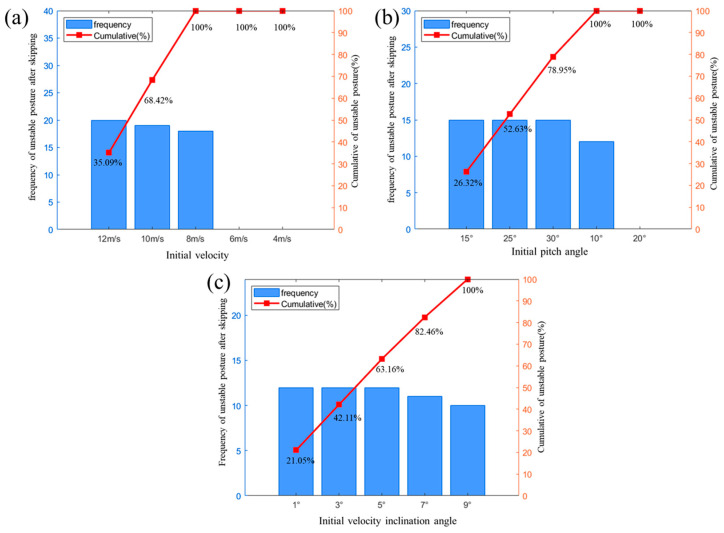
Pareto diagram showing the influence of different motion parameters on motion stability: (**a**) initial velocity; (**b**) initial pitch angle; (**c**) initial velocity inclination angle.

To further investigate the underlying mechanisms of unstable gliding dynamics, a Pareto analysis was conducted of the comprehensive simulation data, with the results presented in Figure 17. The Pareto diagram quantitatively demonstrates that an increased entry velocity and altered pitch angles constitute the primary drivers of unstable gliding behavior. In contrast, the water-entry angle shows a minimal impact, confirming its negligible role in stability deterioration. These findings reveal a critical velocity–pitch coupling effect, where high-speed conditions amplify the destabilizing influence of pitch variations. This mechanistic understanding provides crucial guidelines for establishing stability boundaries in bionic webbed-foot operation.

## 5. Summary

In this study, the CFD method was used to systematically investigate the process of a swan’s webbed foot gliding into water, revealing the dynamic mechanism of the bionic webbed-foot gliding process, which provides a theoretical basis and technical reference for the optimization of the cross-medium vehicle’s water-entry performance. Firstly, characteristic parameters of the swan webbed foot were extracted using reverse engineering methods, enabling the parametric design of the bionic webbed foot. Secondly, computational domain boundary conditions were appropriately set, and mesh independence was verified through grid partitioning, ensuring the reliability of the numerical simulation method. Finally, based on the RANS equations and GMM technology, numerical simulations of the six-degrees-of-freedom bionic webbed-foot gliding process were conducted, with in-depth analysis of flow-field characteristics and time-varying patterns of impact loads, displacement, velocity, and the pitch angle, yielding the following main conclusions:

(1) The near-surface gliding motion of the bionic webbed foot can be divided into two typical stages: stable skipping and surface gliding. In the stable skipping stage, the impact load, vertical displacement, and horizontal velocity of the bionic webbed foot show significant periodic fluctuations. In this process, the bionic webbed foot is subjected to a high impact load when impacting the water, a localized high-pressure zone is formed in the contact area of the water body, and the kinetic energy is transferred to the water body in the form of pressure and gradually dissipated. During the water surface-gliding stage, the webbed foot is continuously subjected to the action of water body gliding resistance, and the speed shows a rapid decay trend. The kinetic mechanism of the bionic webbed foot’s water-gliding behavior can be summarized as follows: Through the progressive gliding resistance of the water body to the bionic flipper, the kinetic energy of the body is gradually dissipated, which avoids the situation of a high impact load due to the instantaneous release of energy when directly impacting with the water. This movement mechanism not only effectively reduces the peak impact load but also significantly improves the stability of the movement process.
(2) The water-entry velocity significantly affects the motion pattern and kinetic characteristics of the bionic webbed foot’s gliding. When the water-entry velocity is lower than 6 m/s, the impact load is not enough to support the flippers to complete the gliding jump, so they show direct gliding into the water. When the water-entry velocity exceeds 8 m/s, the vertical impact load increases, and the phenomenon of gliding into the water after skipping occurs. With the increase in velocity, the bionic webbed foot is subjected to an enhanced impact load and leaves the water faster, which is manifested in an increase in the stabilized skipping and a prolongation of the distance of skipping.
(3) The water-entry pitch angle mainly affects the motion attitude of the bionic webbed foot. As the pitch angle increases, the horizontal impact load increases while the vertical impact load decreases, and the pitching moment changes from positive to negative. When the pitch angle is 20°, the pitching moment amplitude is the smallest, and the motion attitude is the most stable. When the pitch angle is less than 15° or more than 25°, it causes the webbed foot to raise or lower its head rapidly, which is not conducive to stabilizing the slip-and-jump motion and may lead to vehicle motion instability or hitting the water, respectively.
(4) The water-entry velocity angle has a significant effect on the dynamics of the first impact entry. It is shown that the increase in the water-entry velocity angle will lead to an increase in the vertical velocity component, which will aggravate the impact of the water body on the webbed foot, as evidenced by a significant increase in the peak value of the impact load and the amplitude of the pitching moment during the first water entry. Therefore, a smaller water-entry velocity angle is more favorable to enhance the load reduction effect and motion stability of water gliding. This finding provides an important basis for the optimal design of the bionic webbed-foot motion parameters, indicating that more efficient kinetic energy dissipation and a more stable gliding motion can be achieved by reasonably controlling the water-entry velocity angle.

In future research, we will expand our investigation into the water-entry motion of the swan’s webbed foot, focusing on two key aspects: First is the interaction mechanism between bilateral webbed feet. While the current study analyzed the hydrodynamic characteristics of single-webbed-foot water entry, subsequent work will systematically examine the mutual influences of paired webbed feet. Second is the elastic effects of the webbed foot. Presently modeled as rigid bodies, we will further investigate how rigid–flexible coupling properties affect impact load reduction during water entry. These extensions will bridge the gap between single-foot mechanics and whole-body biomechanical strategies, offering potential applications for bio-inspired amphibious robotics.

## Figures and Tables

**Table 1:** Parameters of the swan.

Main Parameters	Symbols	Units	Quantitative Values
Length of the swan’s body	L	mm	810
Width of the swan’s body	D	mm	260
Length of the swan’s webbed foot	l	mm	160
Width of the swan’s webbed foot	d	mm	170
Weight of the swan	M	kg	8
Moment of inertia of the swan about the z-axis	I_ZZ_	kg·m^2^	0.502
Initial pitch angle of the swan’s webbed foot	α_0_	°	variable

**Table 2:** Stable skipping frequency and distance of bionic webbed feet at different water-entry velocities.

Entry Velocity (m/s)	Skipping Frequency	Skipping Distance (m)
8 m/s	3	4.49
10 m/s	5	8.81
12 m/s	8	15.37

**Table 3:** Stable skipping frequency and hydroplaning distance of bionic webbed foot at different water-entry velocity angles.

Entry Velocity Angle (°)	Skipping Frequency	Skipping Distance (m)
1°	3	4.83
3°	3	4.63
5°	3	4.49
7°	2	3.65
9°	2	3.10

## Data Availability

The data presented in this study are available on request from the corresponding author.

## Supplementary Materials

The following supporting information can be downloaded at https://www.bilibili.com/video/BV1yw4m1m7o5/?spm_id_from=333.337.search-card.all.click&vd_source=57ee21a013b32414c0e416a035044354, Video S1: The swan gracefully lands on the water, showing that the key is to push back and open the foot brake.

## Abbreviations

The following abbreviations are used in this manuscript:

CMV	Cross-Medium Vehicle
CFD	Computational Fluid Dynamics
GMM	Global Motion Mesh
UAV	Unmanned Aerial Vehicle
RANS	Reynolds-Averaged Navier–Stokes
VOF	Volume of Fluid
6DOF	Six Degrees of Freedom

## References

[1] Xie H., Jin Y., Bi Y., Zeng Z. (2025). Nezha-D: Dynamic Characteristics and Design of a Ducted HAUV. J. Intell. Robot. Syst..

[2] Yao G., Li Y., Zhang H., Jiang Y., Wang T., Sun F., Yang X. (2023). Review of hybrid aquatic-aerial vehicle (HAAV): Classifications, current status, applications, challenges and technology perspectives. Prog. Aerosp. Sci..

[3] Zeng Z., Lyu C., Bi Y., Jin Y., Lu D., Lian L. (2022). Review of hybrid aerial underwater vehicle: Cross-domain mobility and transitions control. Ocean Eng..

[4] Jin Y., Bi Y., Lyu C., Bai Y., Zeng Z., Lian L. (2024). Nezha-IV: A hybrid aerial underwater vehicle in real ocean environments. J. Field Robot..

[5] Lu M., Tu M., Liao F., Wu S., Xing B., Fan Z., Su Y., Wu W. (2025). NDO-enhanced adaptive fixed-time prescribed performance sliding mode tracking control for coaxial cross-domain flying buoys under unknown ocean disturbances. Ocean Eng..

[6] Li W., Wu W., Miao Q., Wu J. (2025). Research on the Overall Design and Water Entry Simulation of Cross-Media Unmanned Underwater Vehicles. J. Mar. Sci. Eng..

[7] Liu X., Tan L., Zhang X., Li L. (2024). Research of Slamming Load Characteristics during Trans-Media Aircraft Entry into Water. Drones.

[8] Peng T., Peng Y., Sun P., Liu N., Li S. (2024). Mitigating impact loads during water entry by utilizing the air-spring effect. Ocean Eng..

[9] Chen Y., Ren T., Wei J. (2024). Launch characteristics of autonomous underwater vehicle into water and suction characteristics of tail propeller in near free liquid position. Phys. Fluids.

[10] Liu Y., Lei M., Xu H., Liu T., Tian B. (2024). Structure Parameter Effect on Skipping Movement Characteristics of Trans-Media Aircraft Near-Water Surface. Trans. Beijing Inst. Technol..

[11] Gao Y., Zhang H., Li G., Zhou M., Yin H., Gulliver T.A. (2024). Analysis of trans-domain motion process of bullet-shaped trans-domain amphibious vehicle. J. Field Robot..

[12] Clifton G.T., Hedrick T.L., Biewener A.A. (2015). Western and Clark’s grebes use novel strategies for running on water. J. Exp. Biol..

[13] Sharker S.I., Holekamp S., Mansoor M.M., Fish F.E., Truscott T.T. (2019). Water entry impact dynamics of diving birds. Bioinspir. Biomim..

[14] Whitehead J.G., Worrell T., Socha J.J. (2023). Mallard landing behavior on water follows a-constant braking strategy. J. Exp. Biol..

[15] Guo D., Bacciaglia A., Simpson M., Bil C., Marzocca P. Design and development a bimodal unmanned system. Proceedings of the AIAA Scitech 2019 Forum.

[16] Wang X., Zhao J., Pei X., Wang T., Hou T., Yang X. (2024). Bioinspiration review of Aquatic Unmanned Aerial Vehicle (AquaUAV). Biomim. Intell. Robot..

[17] Young P. (2008). Swan.

[18] Mclean C.J., Brassey C.A., Seiter M., Garwood R.J., Gardiner J.D. (2024). The kinematics of amblypygid (Arachnida) pedipalps during predation: Extreme elongation in raptorial appendages does not result in a proportionate increase in reach and closing speed. J. Exp. Biol..

[19] Gibbs B.J., Akanyeti O., Liao J.C. (2024). Kinematics and muscle activity of pectoral fins in rainbow trout (Oncorhynchus mykiss) station holding in turbulent flow. J. Exp. Biol..

[20] Menzer A., Ren Y., Guo J., Tobalske B.W., Dong H. (2022). Wing kinematics and unsteady aerodynamics of a hummingbird pure yawing maneuver. Biomimetics.

[21] Sharma D., Erriguible A., Amiroudine S. (2017). Numerical modeling of the impact pressure in a compressible liquid medium: Application to the slap phase of the locomotion of a basilisk lizard. Theor. Comput. Fluid Dyn..

[22] Wang Z., Peng W., Zhang B. (2024). Kinematics Analysis and Gait Study of Bionic Turtle Crawling Mechanism. Biomimetics.

[23] Chen G., Wei N., Li J., Lu H. (2022). Design and simulation analysis of a bionic ostrich robot. Biomech. Model. Mechanobiol..

[24] Huang J., Liang J., Yang X., Chen H., Wang T. (2021). Cormorant Webbed-feet Support Water-surface Takeoff: Quantitative Analysis via CFD. J. Bionic Eng..

[25] Wang T., Yang X., Liang J., Yao G., Zhao W. (2013). CFD based investigation on the impact acceleration when a gannet impacts with water during plunge diving. Bioinspir. Biomim..

[26] Hou T., Yang X., Wang T., Liang J., Li S., Fan Y. (2020). Locomotor transition: How squid jet from water to air. Bioinspir. Biomim..

[27] Deng J., Zhang L., Liu Z., Mao X. (2019). Numerical prediction of aerodynamic performance for a flying fish during gliding flight. Bioinspir. Biomim..

[28] Dong Y., Liang J., Yang X., Huang J., Xue X., Fan Y. Modeling and simulation of cormorant’s webbed-feet assisted take-off from water surface. Proceedings of the 2017 IEEE International Conference on Robotics and Biomimetics (ROBIO).

[29] Ao P., Wang X., Meng F., Batbayar N., Moriguchi S., Shimada T., Koyama K., Park J., Kim H., Ma M. (2020). Migration routes and conservation status of the Whooper Swan Cygnus cygnus in East Asia. Wildfowl.

[30] Guay P.J., Lorenz R.D., Robinson R.W., Symonds M.R., Weston M.A. (2013). Distance from water, sex and approach direction influence flight distances among habituated black swans. Ethology.

[31] Hirt C.W., Nichols B.D. (1981). Volume of fluid (VOF) method for the dynamics of free boundaries. J. Comput. Phys..

[32] Jones W.P., Launder B.E. (1972). The prediction of laminarization with a two-equation model of turbulence. Int. J. Heat Mass Transf..

[33] Worthy J., Sanderson V., Rubini P. (2001). Comparison of modified k-ε turbulence models for buoyant plumes. Numer. Heat Transf. Part B Fundam..

[34] Sarkar S., Lakshmanan B. (1991). Application of a Reynolds stress turbulence model to the compressible shear layer. AIAA J..

[35] Qu Q., Hu M., Guo H., Liu P., Agarwal R.K. (2015). Study of ditching characteristics of transport aircraft by global moving mesh method. J. Aircr..

[36] Schetz J.A., Bowersox R.D. (2011). Boundary Layer Analysis.

[37] Rosellini L., Hersen F., Clanet C., Bocquet L. (2005). Skipping stones. J. Fluid Mech..

